# Efficacy of Internet‐Based Cognitive Behavioural Therapy (iCBT) on Pain Symptoms and Sleep Quality Improvement in Chronic Pain Patients: A Meta‐Analysis of Randomised Controlled Trials

**DOI:** 10.1002/nop2.70539

**Published:** 2026-05-21

**Authors:** Yuxin Liu, Hong Ji, Sai wei Bai, Xueqing Tian, Wenzhong Zhang, Guo an Jia

**Affiliations:** ^1^ Shandong First Medical University Jinan China; ^2^ The First Affiliated Hospital of Shandong First Medical University Jinan China

## Abstract

**Background:**

Chronic pain is defined as pain that persists or recurs for more than 3 months, involving complex pathological mechanisms and affecting a significant global population. In recent years, Internet‐Based Cognitive Behavioural Therapy (iCBT) has garnered increasing attention as a treatment modality, particularly for alleviating chronic pain and improving sleep quality.

**Design:**

A systematic review and meta‐analysis.

**Methods:**

Comprehensive literature searches were conducted in English databases including PubMed, Web of Science (WoS) Core Collection, Cochrane Library, and Embase, as well as Chinese databases including China National Knowledge Infrastructure (CNKI), Wanfang Data Knowledge Service Platform (Wanfang), and VIP Information from their inception to December 2, 2024. Two independent researchers performed the literature search and data extraction. All statistical analyses were performed using RevMan version 5.

**Results:**

Nine randomised controlled trials were included in the analysis. Following the intervention, iCBT significantly alleviated pain (9 studies, SMD = −0.24, 95% CI = −0.37 to −0.12, *I* ^2^ = 33%). Five studies included long‐term follow‐up, and the results indicated no statistically significant differences between the intervention and control groups (5 studies, SMD = 0.05, 95% CI = −0.10 to 0.19, *I* ^2^ = 0%). Five studies compared post‐intervention and follow‐up results in the intervention group, showing no statistically significant differences (5 studies, SMD = −0.27, 95% CI = −0.73 to 0.19, *I* ^2^ = 88%). Regarding sleep, following the intervention, iCBT significantly enhanced sleep quality (9 studies, SMD = −0.42, 95% CI = −0.71 to −0.14, *I* ^2^ = 76%). Subgroup analysis of sleep outcomes revealed that iCBT was effective for both patients with diagnosed sleep disorders and those without a diagnosis of insomnia. iCBT was effective among patients with diagnosed insomnia (2 studies, SMD = −0.61, 95% CI = −1.17 to −0.04, *I* ^2^ = 70%) and those without a diagnosis of insomnia (7 studies, SMD = −0.37, 95% CI = −0.66 to −0.07, *I* ^2^ = 67%). iCBT was also found to be effective in adults aged ≥ 18 years (6 studies, SMD = −0.71, 95% CI = −0.87 to −0.54, *I* ^2^ = 29%). Additionally, subgroup analysis of intervention delivery modes showed hospital web‐based self‐administered (2 studies, SMD = −0.83, 95% CI = −1.17 to −0.50, *I* ^2^ = 0%) and synchronous remote interventions (2 studies, SMD = −0.79, 95% CI = −1.01 to −0.57, *I* ^2^ = 0%) significantly improved sleep quality.

**Conclusion:**

iCBT is an effective intervention for the management of chronic pain, significantly alleviating pain and improving sleep quality in patients. It is particularly effective in enhancing sleep quality in adults aged ≥ 18 years.

**Trial Registration:**

PROSPERO identifier: CRD42025634381

## 1 Introduction

Chronic pain is defined as pain that persists or recurs for more than 3 months. In contrast to acute pain, chronic pain involves more complex pathological mechanisms (‘Definitions of Chronic Pain Syndromes’, n.d.). Chronic pain encompasses diverse types, such as chronic back pain, cancer pain, and orthopaedic‐related pain, and represents a common and multifaceted global health issue. Globally, the prevalence of chronic pain ranges from 20% to 30%, with significant variations across regions and populations. For example, in 2021, the prevalence of chronic pain among adults in the United States was 20.9% (Rikard et al. 2023). A systematic review by Rometsch et al. (2025) reported that the prevalence of chronic pain in Europe varies widely, ranging from 12% to 48%. In China, the overall prevalence of chronic pain is 31.54%, while it rises to 60.02% among the elderly population (Ai et al. 2023; Zheng et al. 2020).

These statistics highlight the global prevalence of chronic pain, which extends beyond a physiological issue and often co‐occurs with mental health conditions such as insomnia and depression. Sleep disturbances are common in patients with chronic pain; for example, 52% of those with chronic back pain report such issues (Tang et al. 2007). A meta‐analysis found that 75.3% of non‐cancer pain patients experienced sleep disturbances, and 72.9% met the criteria for clinical insomnia (Sun et al. 2021). Chronic pain not only affects patients' health but also has significant social and economic consequences. It increases healthcare costs due to the need for long‐term management, particularly in developing countries with limited resources (Kawai et al. 2017). Moreover, chronic pain leads to productivity losses, especially among working‐age individuals, impacting daily activities and work capacity, which adds to the economic burden on society. Thus, chronic pain poses a dual burden on both physical and mental health, making it a long‐term social and economic challenge. Effective interventions, particularly those addressing sleep issues in patients with chronic pain, are urgently needed to mitigate these impacts.

Currently, the treatment of chronic pain primarily relies on medications, especially non‐steroidal anti‐inflammatory drugs (NSAIDs), analgesics, and antidepressants, which are effective in relieving pain, particularly for patients with acute and moderate pain (Birkinshaw et al. 2024; Moulin et al. 2014). However, prolonged use of these medications can lead to side effects, such as gastrointestinal discomfort, kidney damage, cardiovascular risks, and drug dependence (Haines et al. 2023). Additionally, some patients, concerned about the addictive potential of these drugs, may discontinue treatment once pain relief is achieved, which leads to poor medication adherence and compromised treatment outcomes. Cognitive Behavioural Therapy (CBT) is a structured, time‐limited, and goal‐oriented therapeutic approach. Its core principle is that emotions and behaviours are shaped by individual cognitions (thoughts and beliefs), and by modifying maladaptive cognitive patterns, CBT can improve emotional and behavioural outcomes (Beck 1991). As an emerging non‐pharmacological intervention, CBT has shown promising results in alleviating chronic pain, enhancing sleep quality, and addressing pain‐related psychological issues (He et al. 2023; Selvanathan et al. 2021). By helping patients modify their cognitive and emotional responses to pain, CBT can significantly reduce pain perception and demonstrate substantial benefits in alleviating pain‐related sleep disturbances. Compared to pharmacological interventions, CBT offers greater flexibility and durability, with no associated adverse reactions or side effects (Selvanathan et al. 2021).

CBT, a non‐pharmacological treatment, has been widely validated for its effectiveness in alleviating chronic pain and improving sleep quality. With the rapid development of technology, Internet‐Based Cognitive Behavioural Therapy (iCBT) has emerged as an important tool in the management of chronic pain. Compared to traditional face‐to‐face CBT, iCBT offers treatment through an online platform or computer, enabling patients to receive interventions from the comfort of their homes, making it particularly suitable for those who cannot visit healthcare providers frequently due to geographical, scheduling, or health limitations (Cervin et al. 2024). iCBT uses innovative technological methods to offer personalised, home‐based treatment, overcoming the time and space limitations of traditional face‐to‐face therapy while enhancing accessibility and flexibility (Gandy et al. 2022).

With the rise of the ‘nursing + Internet’ model, iCBT has evolved significantly—from traditional telephone interventions to modern internet platforms and mobile applications. These technological advancements have expanded the accessibility and popularity of CBT. As iCBT methods have developed, research has increased, with studies incorporating iCBT into chronic pain management and sleep improvement. Evidence suggests that internet‐based CBT is as effective as traditional face‐to‐face interventions in alleviating chronic pain and improving sleep quality (Hurley‐Wallace et al. 2021). Randomised controlled trials further support iCBT as a promising alternative for long‐term pain management (Mehta et al. 2019). However, many existing studies still have limitations, including small sample sizes and high heterogeneity in evaluating pain relief and sleep quality. This study aims to conduct a meta‐analysis to systematically assess iCBT's effectiveness in improving pain symptoms and sleep quality in patients with chronic pain, providing stronger evidence for clinical practice.

## 2 Methods

### 2.1 Search Strategy

A comprehensive search was performed in English databases, including PubMed, Web of Science (WoS) Core Collection, Cochrane Library, and Embase, as well as Chinese databases including China National Knowledge Infrastructure (CNKI), Wanfang Data Knowledge Service Platform (Wanfang), and VIP Information (VIP, formerly known as Chongqing VIP Information Co. Ltd.) from the inception of each database to December 2, 2024. The search terms were a combination of Medical Subject Headings (MeSH) and keywords related to iCBT and chronic pain. The search terms were as follows: (Web‐assisted OR Telephone OR Web‐based OR Internet‐Based OR Online OR Computer‐based OR Website OR Smartphone OR Mobile Application OR mHealth OR eHealth OR Telehealth) AND (Cognitive Behavioural Therapy OR CBT‐I OR Internet‐Based Cognitive Behavioural Therapy (iCBT) OR Online Cognitive Behavioural Therapy) AND (chronic pain OR chronic pain* OR pain). The detailed search strategies for each database are presented in Appendix 1. The search terms were selected to identify randomised controlled trials (RCTs) on the application of Internet‐Based Cognitive Behavioural Therapy (iCBT) for chronic pain patients. Additionally, we manually reviewed the reference lists of the included high‐relevance studies to identify any additional eligible trials.

### 2.2 Eligibility Criteria

The literature search was restricted to studies published in English or Chinese. Eligible studies had to meet the following inclusion criteria: (a) *Participants*: The target population consisted of patients with chronic pain such as postoperative pain, bone pain, etc., with no restrictions on the types of chronic pain. (b) *Interventions*: Studies that employed iCBT (e.g., via phone, video, computer, etc.) in the intervention group were included. The cognitive behavioural interventions in the included studies could include specialised applications, such as Cognitive Behavioural Therapy for Insomnia (CBT‐I) or Cognitive Behavioural Therapy for Depression (CBT‐D). (c) Control: Interventions in the control group were categorised into three types for clarity: ① Standard care; ② Specific treatments (including in‐person cognitive behavioural therapy, specialised headache treatment, and relaxation training); ③ Health education. (d) Outcome: The primary or secondary outcomes in the included studies assessed pain and sleep conditions.

### 2.3 Study Selection

All database search results were imported into Zotero for duplicate study deletion and study management. Two independent reviewers (Reviewer A and Reviewer B) screened the titles and abstracts of the studies in accordance with the eligibility criteria. Potentially eligible studies were identified, and final inclusion was determined after re‐screening the full texts. Then, the two reviewers discussed and reached a consensus on the final included studies.

### 2.4 Data Extraction

Two reviewers independently extracted the following data from each included study: publication details (authors, year of study, country), population characteristics (sample size, age, etc.), intervention details (intervention group, control group), main assessment tools, and time points for outcome evaluation. Detailed information is provided in Table 1.

**TABLE 1:** Characteristics of the included studies.

Authors, year	Country	Participants		Intervention methods		Assessment tools in pain and sleep	Outcomes assessment time	Intervention cycles (weeks)	Intervention delivery modes
		Sample size (IG/CG)	Mean Age ± SD (IG/CG)	Intervention group (IG)	Control group (CG)				
Burke et al. (2019)	Ireland	35/34	50.00 ± 12.30/52.00 ± 13.80	iCBT	Standard care	①, B	Postintervention, 3‐month follow‐up	6	a
Fales et al. (2015)	USA	14/17	14.00 ± 6.00/14.00 ± 6.00	iCBT	Standard care	③, C	Postintervention	8–10	a
Gong (2021)	China	36/36	40.36 ± 9.98/39.14 ± 10.82	CCBT	Standard care	③, D	Postintervention	2	b
Law et al. (2015)	USA	44/39	14.60 ± 1.80/14.30 ± 1.60	iCBT	Standard care	②, C	Postintervention, 3‐month follow‐up	8	a
Mariano et al. (2021)	USA	32/22	48.20 ± 11.10/50.60 ± 13.60	iCBT	Standard care	①, A	Postintervention	8	c
McCurry et al. (2021)	USA	136/146	70.10 ± 7.10/70.40 ± 6.50	iCBT	Health education	①, A	Postintervention, 12‐month follow‐up	8	c
Palermo et al. (2016)	USA	138/135	14.63 ± 1.62/14.70 ± 1.72	iCBT	Health education	③, C	Postintervention, 6‐month follow‐up	8–10	a
Wiklund et al. (2022)	Sweden	30/24	48.20 ± 11.10/50.60 ± 13.60	iCBT‐I	Relaxation	③, A	Postintervention, 6‐month follow‐up	5	a
Zhou (2020)	China	40/40	40.15 ± 8.97/39.45 ± 10.76	CCBT	Standard care	③, D	Postintervention	2	b

*Note:* Assessment Tools in pain: ① The Brief Pain Inventory (BPI); ② Total headache days: 11‐point numerical rating scale (NRS) via the prospective 7‐day online diary; ③ Pain intensity: an 11‐point numerical rating scale (NRS) Index. Assessment Tools in sleep: A: Insomnia Severity Index (ISI); B: The Pittsburgh Sleep Quality Index (PSQI); C: Numeric Rating Scale (NRS); D: Athens Insomnia Scale (AIS). Intervention delivery modes: a: General web‐based self‐administered intervention (GWBSAI); b: Hospital web‐based self‐administered intervention (HWBSAI); c: Synchronous remote intervention (SRI).

Abbreviations: CCBT, Computerised Cognitive Behavioural Therapy; CG, Control Group; iCBT, Internet‐Based Cognitive Behavioural Therapy; iCBT‐I, Internet‐Based Cognitive Behavioural Therapy for Insomnia; IG, Intervention Group.

### 2.5 Article Registration

This article has been registered on the PROSPERO platform, with the registration number CRD42025634381. No amendments were made to the original protocol.

## 3 Results

### 3.1 Study Selection

**FIGURE 1:**
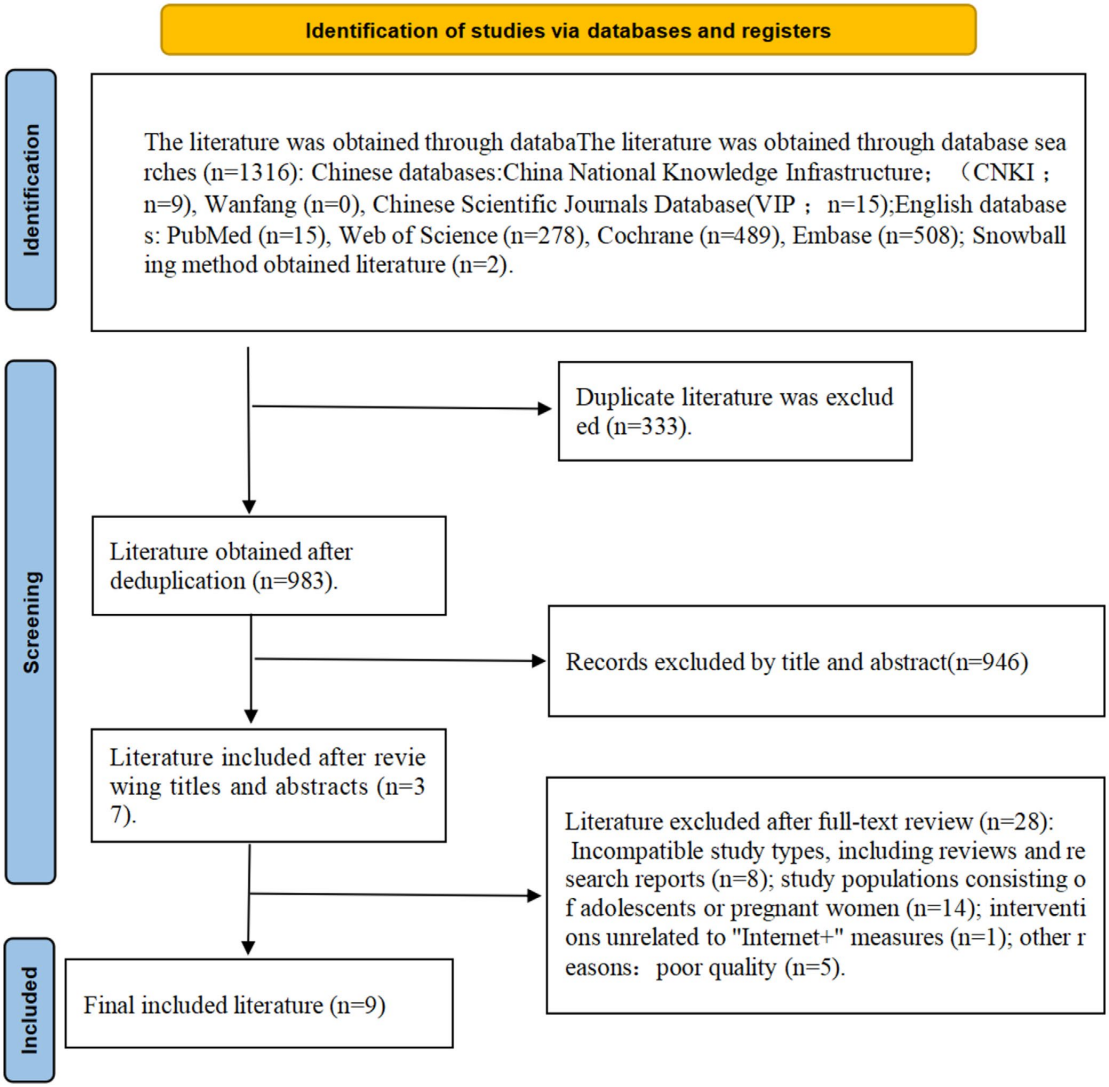
PRISMA flow chart for study selection.

A total of 9 randomised controlled trials were finally included after screening, and the detailed study selection process and exclusion reasons are shown in Figure 1 (PRISMA flowchart). In total, 1316 records were identified via databases and registers, and 333 potentially eligible studies were identified after duplicate records were removed. 37 studies were retrieved and reviewed more thoroughly after screening titles and abstracts. 9 randomised controlled trials met the eligibility criteria. No additional eligible studies were identified after full‐text screening.

### 3.2 Study Characteristics

The characteristics of the included studies (e.g., sample size, intervention details, and assessment tools) are summarised in Table 1. The studies were conducted in China, Ireland, Sweden, and the United States from 2015 to 2022. Sample sizes ranged from 31 to 282, and the ages of the patients varied from 14.30 (SD = 1.60) to 70.10 (SD = 7.10) years. Different assessment tools were used in each study to evaluate pain symptoms and sleep conditions. The pain intensity assessment tools included the Brief Pain Inventory (BPI), total headache days, and pain intensity. The primary assessment tools for sleep conditions included the Pittsburgh Sleep Quality Index (PSQI), Sleep Quality 0 to 10 NRS, Insomnia Severity Index (ISI), and Athens Insomnia Scale (AIS). All nine studies assessed both the intervention and control groups after the intervention, and five studies included follow‐up assessments, with follow‐up durations ranging from 3 to 12 months.

### 3.3 Quality Assessment

**FIGURE 2:**
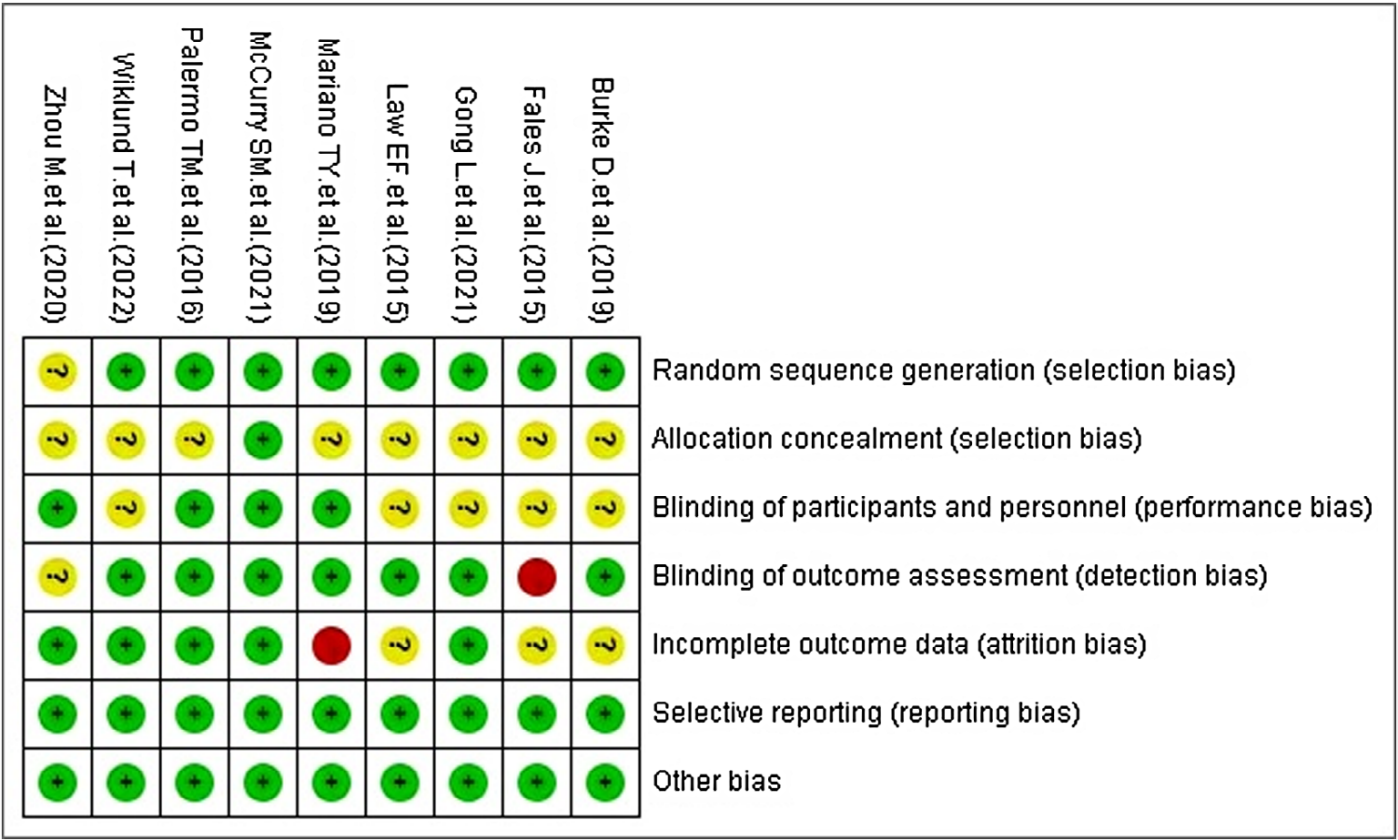
Risk of bias summary: Review authors' judgements about risk of bias item for each included study.

Two independent reviewers assessed the quality of the nine included studies according to the Cochrane RCT quality assessment criteria. One study was rated as Grade A, while the remaining eight studies were rated as Grade B (see Figure 2).

### 3.4 Outcomes From Included Studies

Pooled standardised mean differences (SMDs), 95% confidence intervals, heterogeneity test results, and overall effect sizes for pain and sleep outcomes are presented in Table 2. Subgroup analysis based on control intervention types showed no significant difference in the effect size of iCBT among the three control categories (interaction *p* > 0.05), indicating that the type of control intervention is not a major source of heterogeneity.

**TABLE 2:** Results of the included studies.

Outcomes	*N*	Studies	Pooled SMD (95% CI)	Heterogeneity	Test for overall effect
Pain					
Post‐treatment	9	Burke et al. (2019), Fales et al. (2015), Gong (2021), Law et al. (2015), Mariano et al. (2021), McCurry et al. (2021), Palermo et al. (2016), Wiklund et al. (2022), Zhou (2020)	−0.24 [−0.37, −0.12]	*Q* (df = 8) = 11.90 (*p* = 0.16); *I* ^2^ = 33%	*Z* = 3.80 (*p =* 0.0001)
Follow‐Up	5	Burke et al. (2019), Law et al. (2015), McCurry et al. (2021), Palermo et al. (2016), Wiklund et al. (2022)	0.05 [−0.10, 0.19]	*Q* (df = 4) = 1.62 (*p* = 0.81); *I* ^2^ = 0%	*Z* = 0.60 (*p* = 0.55)
Post‐intervention and follow‐up in Intervention Group	5	Burke et al. (2019), Law et al. (2015), McCurry et al. (2021), Palermo et al. (2016), Wiklund et al. (2022)	−0.27 [−0.73, 0.19]	*Q* (df = 4) = 32.03 (*p* < 0.00001); *I* ^2^ = 88%; *t* ^2^ = 0.23	*Z* = 1.49 (*p* = 0.25)
Sleep					
Sleep	9	Burke et al. (2019), Fales et al. (2015), Gong (2021), Law et al. (2015), Mariano et al. (2021), McCurry et al. (2021), Palermo et al. (2016), Wiklund et al. (2022), Zhou (2020)	−0.42 [−0.71, −0.14]	*Q* (df = 8) = 33.11 (*p* < 0.00001); *I* ^2^ = 76%; *t* ^2^ = 0.13	*Z* = 2.94 (*p* = 0.003)
Sleep Subgroup analysis					
Diagnosed insomnia	2	McCurry et al. (2021), Wiklund et al. (2022)	−0.61 [−1.17, −0.04]	*Q* (df = 1) = 3.36 (*p* = 0.07); *I* ^2^ = 70%; *t* ^2^ = 0.12	*Z* = 2.10 (*p* = 0.04)
Undiagnosed insomnia	7	Burke et al. (2019), Fales et al. (2015), Gong (2021), Law et al. (2015), Mariano et al. (2021), Palermo et al. (2016), Zhou (2020)	−0.37[−0.66, −0.07]	*Q*(df = 6) = 18.34 (*p* = 0.005); *I* ^2^ = 67%, *t* ^2^ = 0.10	*Z* = 2.4 (*p* = 0.02)
Age ≥ 18	6	Burke et al. (2019), Gong (2021), Mariano et al. (2021), McCurry et al. (2021), Wiklund et al. (2022), Zhou (2020)	−0.71[−0.87, −0.54]	*Q*(df = 5) = 7.02 (*p* = 0.22); *I* ^2^ = 29%	*Z* = 8.36 (*p* < 0.00001)
Age < 18	3	Fales et al. (2015), Law et al. (2015), Palermo et al. (2016)	−0.03 [−0.23, 0.18]	*Q*(df = 2) = 0.08 (*p* = 0.96); *I* ^2^ = 0%	*Z* = 0.25 (*p* = 0.8)
Insomnia Severity Index (ISI)	3	Mariano et al. (2021), McCurry et al. (2021), Wiklund et al. (2022)	−0.72[−0.93, −0.51]	*Q*(df = 2) = 3.88 (*p* = 0.14); *I* ^2^ = 48%	*Z* = 6.78 (*p* < 0.00001)
Athens Insomnia Scale (AIS)	2	Gong (2021), Zhou (2020)	−0.83[−1.17, −0.50]	*Q*(df = 1) = 0.61 (*p* = 0.43); *I* ^2^ = 0%	*Z* = 4.91 (*p* < 0.00001)
Numeric Rating Scale (NRS)	3	Fales et al. (2015), Law et al. (2015), Palermo et al. (2016)	−0.04[−0.25, 0.16]	*Q*(df = 2) = 0.09 (*p* = 0.96); *I* ^2^ = 0%	*Z* = 0.43 (*p* = 0.67)
Intervention delivery modes					
General web‐based self‐administered intervention	5	Burke et al. (2019), Fales et al. (2015), Law et al. (2015), Palermo et al. (2016), Wiklund et al. (2022)	−0.09[−0.27, 0.08]	*Q* (df = 4) = 2.06 (*p* = 0.73); *I* ^2^ = 0%	*Z* = 1.04 (*p* = 0.30)
Hospital web‐based self‐administered intervention (HWBSAI)	2	Gong (2021), Zhou (2020)	−0.83 [−1.17, −0.50]	*Q* (df = 1) = 0.61 (*p* = 0.43); *I* ^2^ = 0%	*Z* = 4.91 (*p* < 0.00001)
Synchronous remote intervention (SRI)	2	Mariano et al. (2021), McCurry et al. (2021)	−0.79 [−1.01, −0.57]	*Q* (df = 1) = 0.99 (*p* = 0.32); *I* ^2^ = 0%	*Z* = 6.94 (*p* < 0.00001)

*Note:* *N*, The number of included studies; *t* ^2^, Abbreviation for Tau (Ai et al. 2023) (heterogeneity variance); *I* ^2^, Abbreviation for *I* ^2^ (heterogeneity index, with interpretation).

### 3.5 Pain Symptoms

#### 3.5.1 Pain Symptoms After Intervention

**FIGURE 3:**
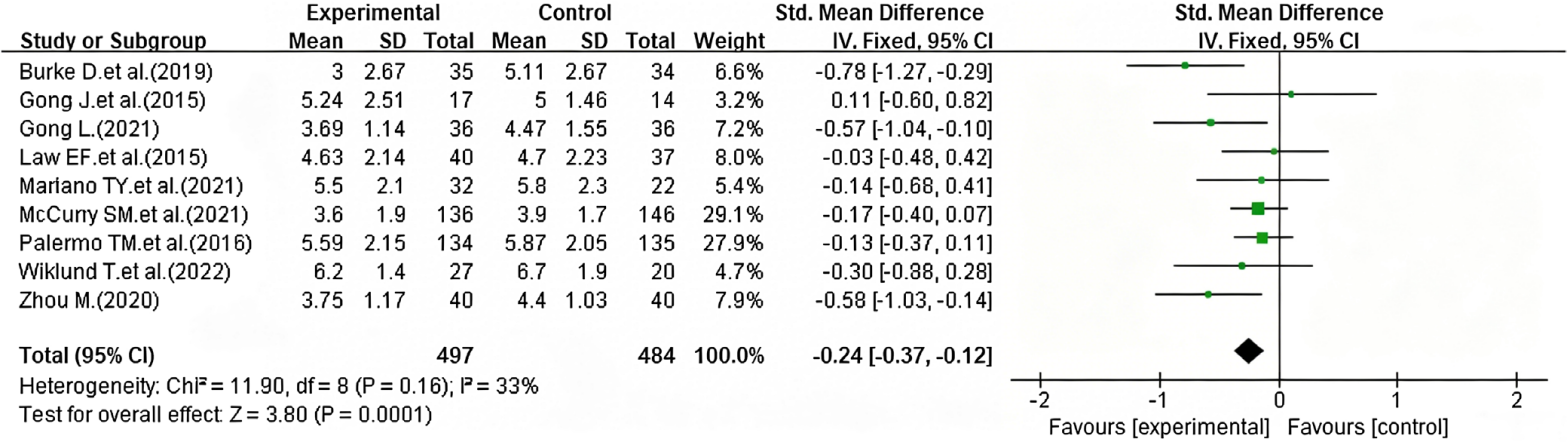
Forest plot of the efficacy of iCBT versus standard care in improving pain symptoms (post‐intervention). The horizontal axis represents the standardised mean difference (*SMD*) for pain symptoms; negative values indicate better pain relief in the iCBT group compared to the control group.

In the nine included studies, five (Fales et al. 2015; Gong 2021; Palermo et al. 2016; Wiklund et al. 2022; Zhou 2020) employed the 0–10 Pain Intensity (NRS) scale, three (Burke et al. 2019; Mariano et al. 2021; McCurry et al. 2021) used the Pain Interference: The Brief Pain Inventory (BPI) scale, and one (Law et al. 2015) used Total Headache Days to assess chronic headache status in children. After the relevant data from the nine studies were imported into the software, heterogeneity between the studies was found to be small, and a fixed‐effect model was applied for analysis. The results indicated that iCBT improves pain symptoms in patients with chronic pain (pooled SMD = −0.24; 95% CI: −0.37 to −0.12, *p* = 0.0001) (Figure 3).

#### 3.5.2 Differences in Pain Symptom Scores After Long‐Term Follow‐Up

**FIGURE 4:**
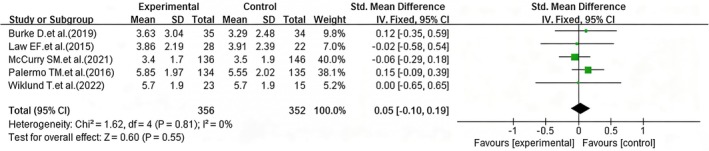
Forest plot of pain improvement effects between the intervention and control groups (post‐intervention vs. follow‐up). The horizontal axis represents the standardised mean difference (*SMD*) for pain symptoms; negative values indicate better pain relief in the iCBT group compared to the control group.

Of the nine included studies, five (Burke et al. 2019; Law et al. 2015; McCurry et al. 2021; Palermo et al. 2016; Wiklund et al. 2022) assessed pain symptom scores after at least 3 months of follow‐up. The data from these five studies were imported into the software, and due to low heterogeneity, a fixed‐effect model was applied. The results indicated that, after long‐term follow‐up, no statistically significant difference was found in pain symptom scores between the intervention and control groups (pooled SMD = 0.05; 95% CI: −0.10 to 0.19, *p* = 0.55) (Figure 4).

#### 3.5.3 The Difference in Pain Before and After Long‐Term Follow‐Up in the Intervention Group

**FIGURE 5:**
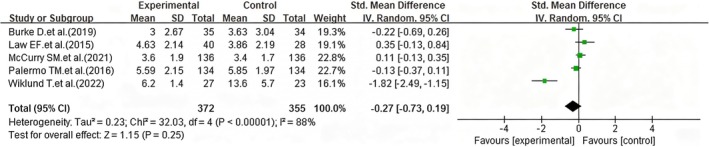
Forest plot of pain improvement effects within the intervention group (post‐intervention vs. follow‐up). The horizontal axis represents the standardised mean difference (*SMD*) for pain symptoms; negative values indicate better pain relief at follow‐up compared to post‐intervention in the iCBT group.

Of the nine included studies, five (Burke et al. 2019; Law et al. 2015; McCurry et al. 2021; Palermo et al. 2016; Wiklund et al. 2022) with follow‐up data were analysed in the meta‐analysis. The results revealed substantial heterogeneity between the studies, necessitating the use of a random‐effects model for the meta‐analysis. The results indicated that the difference in pain symptom scores before and after long‐term follow‐up in the intervention group was not statistically significant (*SMD* = −0.27, 95% CI: −0.73 to 0.19, *p* = 0.25) (Figure 5), suggesting that its long‐term effectiveness may be limited.

### 3.6 Sleep Conditions

#### 3.6.1 Overall Effect of iCBT on Sleep Quality Improvement

**FIGURE 6:**
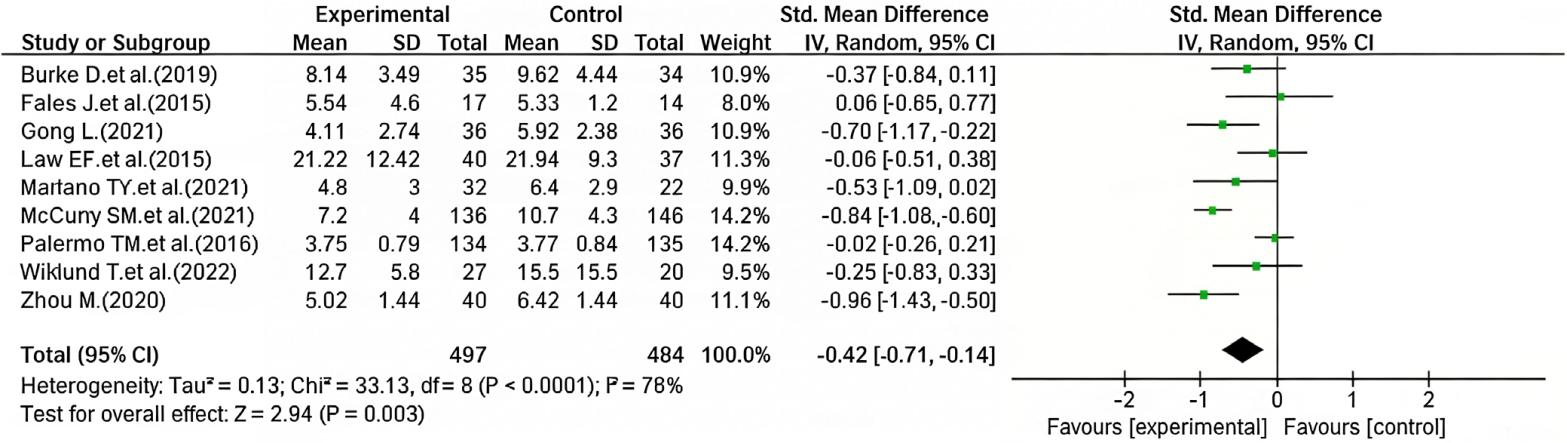
Forest plot of the efficacy of iCBT versus control in improving sleep quality (post‐intervention). The horizontal axis represents the standardised mean difference (*SMD*) for sleep quality; negative values indicate better sleep quality in the iCBT group compared to the control group.

A statistically significant difference was observed in sleep quality improvement between the iCBT group and the control group. Of the nine included studies, three (Mariano et al. 2021; McCurry et al. 2021; Wiklund et al. 2022) employed the Insomnia Severity Index (ISI) scale; two studies (Gong 2021; Zhou 2020) employed the Athens Insomnia Scale (AIS), three articles (Fales et al. 2015; Law et al. 2015; Palermo et al. 2016) used a 0–10 sleep quality rating scale, and one (Burke et al. 2019) used the Pittsburgh Sleep Quality Index (PSQI). The heterogeneity between the included studies was substantial, necessitating the use of a random‐effects model for the meta‐analysis. The results indicated that the effect of iCBT on sleep improvement was significantly different from that of the control group (SMD = −0.42, 95% CI: −0.71 to −0.14, *p* = 0.003) (Figure 6).

#### 3.6.2 Sleep Subgroup Analysis

Subgroup analyses were performed to explore the sources of substantial heterogeneity in the effect of iCBT on sleep quality improvement in patients with chronic pain, with stratification by insomnia diagnosis status, sleep quality assessment tools, age groups, and intervention delivery modes. The detailed results are as follows:

##### 3.6.2.1 Diagnosed With Insomnia

**FIGURE 7:**
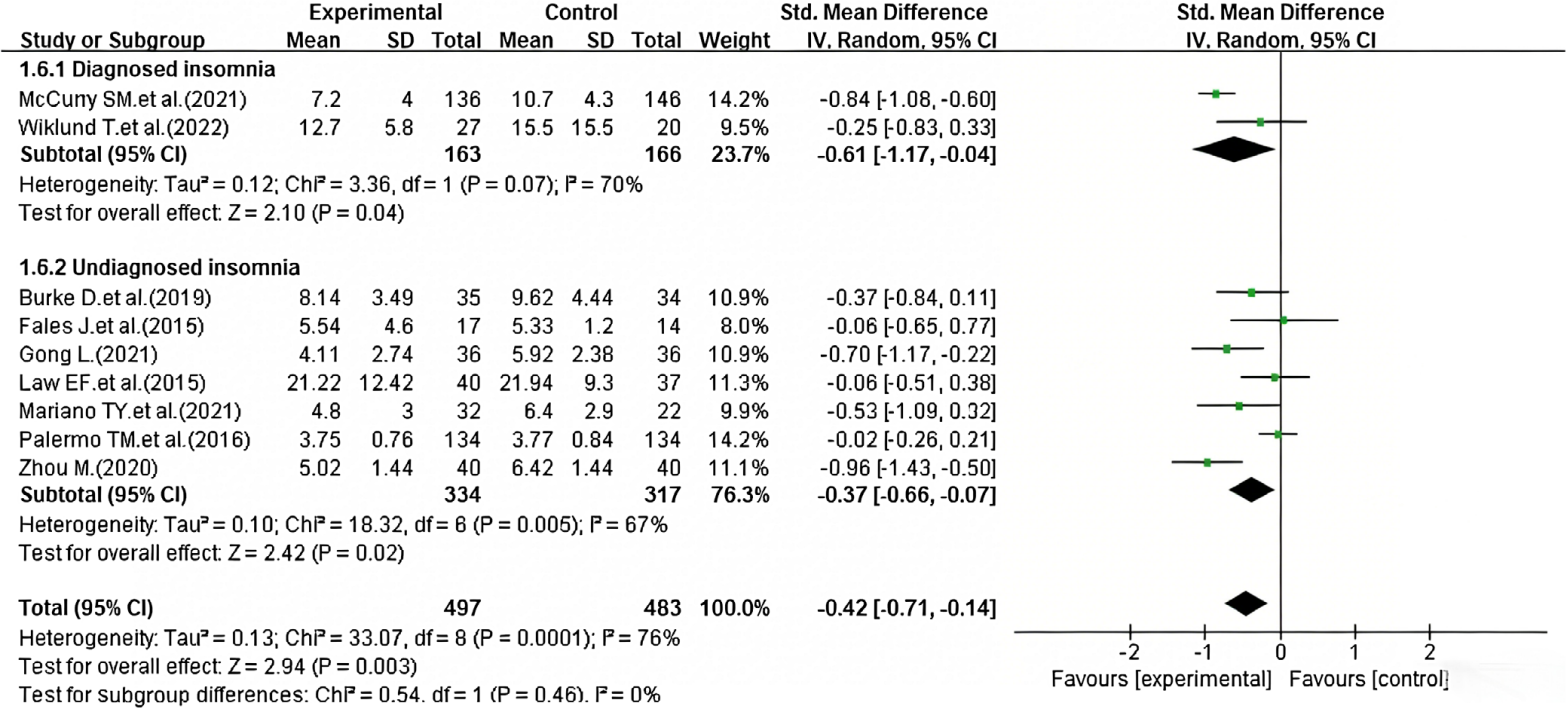
Forest plot of subgroup analysis: Efficacy of iCBT on sleep quality by insomnia diagnosis status. The horizontal axis represents the standardised mean difference (*SMD*) for sleep quality; negative values indicate better sleep quality in the iCBT group compared to the control group.

Heterogeneity was observed in the effect of iCBT on sleep quality improvement in patients with chronic pain, leading to the division of the studies into two subgroups: diagnosed insomnia chronic pain patients and non‐diagnosed insomnia chronic pain patients. The subgroup of chronic pain patients with diagnosed insomnia included two studies (McCurry et al. 2021; Wiklund et al. 2022). The heterogeneity test revealed *I* ^2^ = 70%, *p* = 0.07, indicating substantial heterogeneity. Consequently, a random‐effects model was applied for the meta‐analysis, and the results indicated a statistically significant difference (SMD = −0.61, 95% CI: −1.17 to −0.04, *p* = 0.04) (Figure 7), suggesting that iCBT improved sleep quality in patients with chronic pain and insomnia. The subgroup of chronic pain patients without diagnosed insomnia included seven studies (Burke et al. 2019; Fales et al. 2015; Gong 2021; Law et al. 2015; Mariano et al. 2021; Palermo et al. 2016; Zhou 2020). The heterogeneity test revealed *I* ^2 =^ 67%, *p* < 0.05, indicating moderate heterogeneity. A random‐effects model was applied for the meta analysis, and the results indicated a statistically significant difference (SMD = −0.37, 95% CI: −0.66 to −0.07, *p* = 0.02) (Figure 7). This indicates that iCBT remains effective for chronic pain patients who have not been diagnosed with insomnia. Furthermore, the high residual heterogeneity suggests that the subgroup analysis factor of ‘whether insomnia is diagnosed’ is not the main source of heterogeneity.

##### 3.6.2.2 Sleep Quality Assessment Tools

**FIGURE 8:**
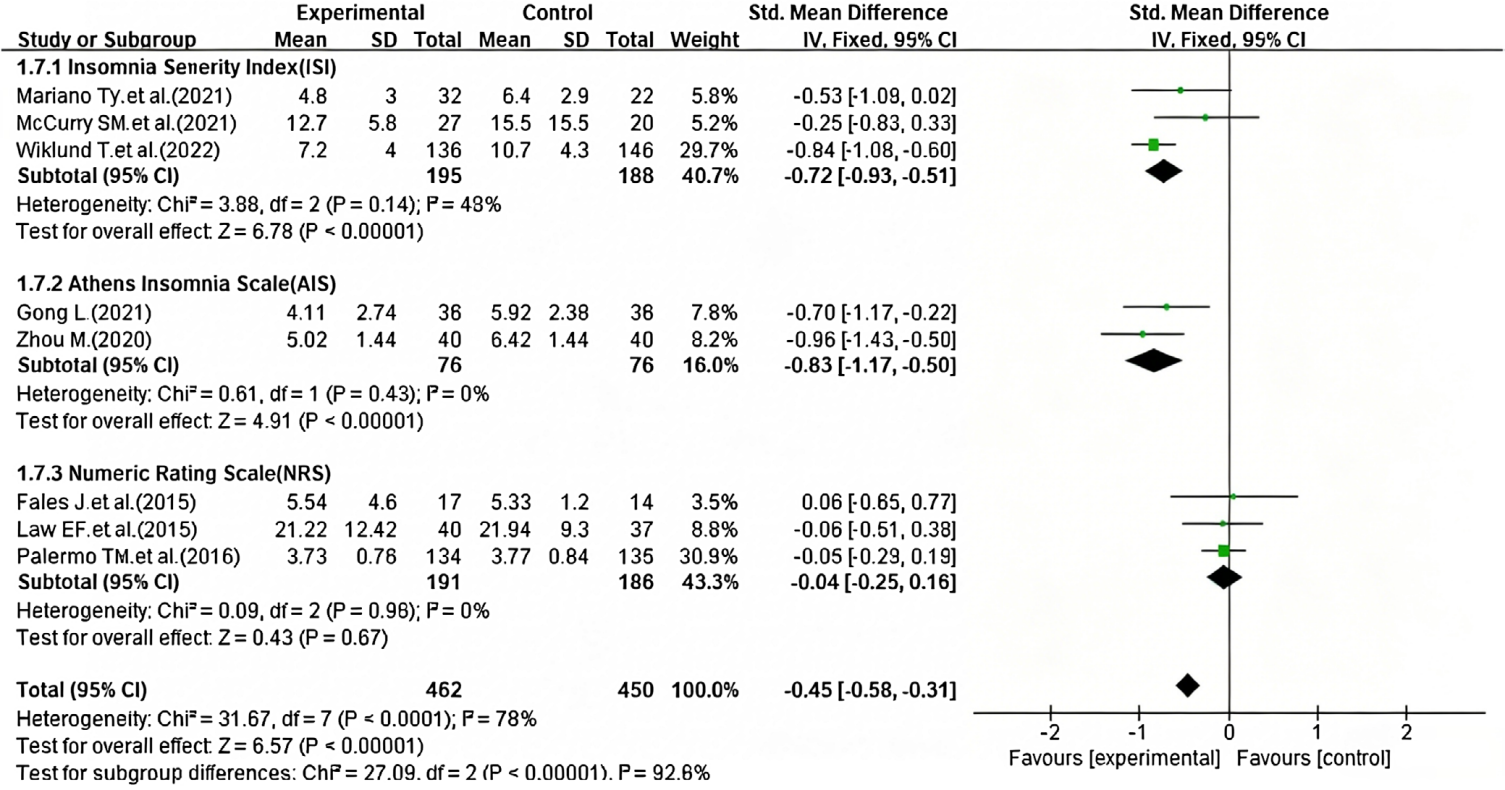
Forest plot of subgroup analysis: Efficacy of iCBT on sleep quality by sleep assessment tools. The horizontal axis represents the standardised mean difference (*SMD*) for sleep quality; negative values indicate better sleep quality in the iCBT group compared to the control group.

In this study, the pooled total effect across all studies showed high heterogeneity (*I* ^2^ = 78%, *p* < 0.0001), prompting subgroup analyses to explore potential sources of heterogeneity. Subgroup analyses were stratified by different sleep quality assessment tools, including the Insomnia Severity Index (ISI), Athens Insomnia Scale (AIS), and Numeric Rating Scale (NRS). The results demonstrated a significant reduction in heterogeneity across the various tools, with the *I* ^2^ value being less than 50%. A fixed‐effect model was applied for the meta‐analysis, suggesting that the choice of assessment tool may represent a crucial factor contributing to this heterogeneity. Specifically, subgroup analyses utilizing the Insomnia Severity Index (ISI) (Mariano et al. 2021; McCurry et al. 2021; Wiklund et al. 2022) (SMD = −0.72, 95% CI: −0.93 to −0.51, *p* < 0.01) (Figure 8), and the Athens Insomnia Scale (AIS) (Gong 2021; Zhou 2020) (SMD = −0.83, 95% CI: −1.17 to −0.50, *p* < 0.01) (Figure 8), yielded statistically significant results, indicating that these tools are sensitive to improvements in sleep quality associated with iCBT. In contrast, subgroup analyses from three studies (Fales et al. 2015; Law et al. 2015; Palermo et al. 2016) employing the Numeric Rating Scale (NRS) failed to show statistical significance (SMD = −0.04, 95% CI: −0.25 to 0.16, *p* = 0.67) (Figure 8), suggesting that the Numeric Rating Scale (NRS) may not be sensitive enough to detect the sleep improvement effects of iCBT.

##### 3.6.2.3 Subgroup Analysis by Age

**FIGURE 9:**
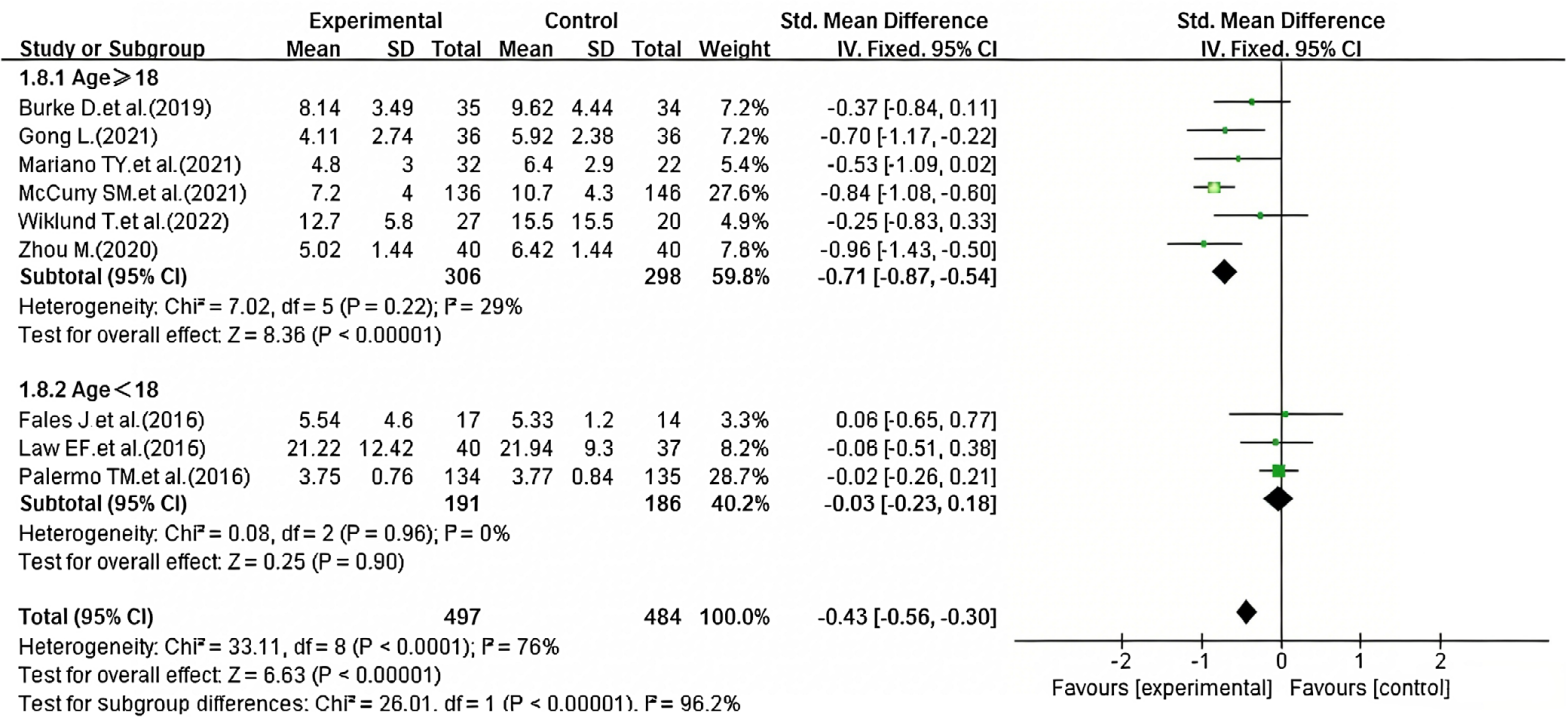
Forest plot of subgroup analysis: Efficacy of iCBT on sleep quality by age groups (≥ 18 years vs. < 18 years). The horizontal axis represents the standardised mean difference (*SMD*) for sleep quality; negative values indicate better sleep quality in the iCBT group compared to the control group.

The pooled effect of iCBT on sleep quality in patients with chronic pain showed high heterogeneity across all included studies (*I* ^2^ = 76%, *p* < 0.0001), thus a random‐effects model was used for the overall meta‐analysis. To explore the sources of this substantial heterogeneity, subgroup analyses were performed by participant age (≥ 18 years vs. < 18 years). Of the nine included studies, three focused on adolescents and children under 18 years old, while the other six (Burke et al. 2019; Gong 2021; Mariano et al. 2021; McCurry et al. 2021; Wiklund et al. 2022; Zhou 2020) involved participants aged ≥ 18 years. A subgroup analysis was performed based on age. For the six studies involving chronic pain patients aged ≥ 18 years, the heterogeneity analysis revealed *I* ^2^ = 29%, indicating low heterogeneity. Consequently, a fixed‐effect model was applied for the meta‐analysis, and the results indicated a statistically significant difference (SMD = −0.71, 95% CI: −0.87 to −0.54, *p* < 0.01) (Figure 9.), suggesting that iCBT improves sleep quality in patients with chronic pain aged ≥ 18 years. However, for patients aged < 18 years old (Fales et al. 2015; Law et al. 2015; Palermo et al. 2016), the meta‐analysis of the intervention and control groups revealed no statistically significant difference (SMD = −0.03, 95% CI: −0.23 to 0.18, *p* = 0.96) (Figure 9), indicating that iCBT did not have a significant effect on sleep quality in this age group. The low heterogeneity in adults aged ≥ 18 years may be due to consistent intervention protocols and similar baseline characteristics across studies.

##### 3.6.2.4 Subgroup Analysis by Intervention Delivery Modes

**FIGURE 10:**
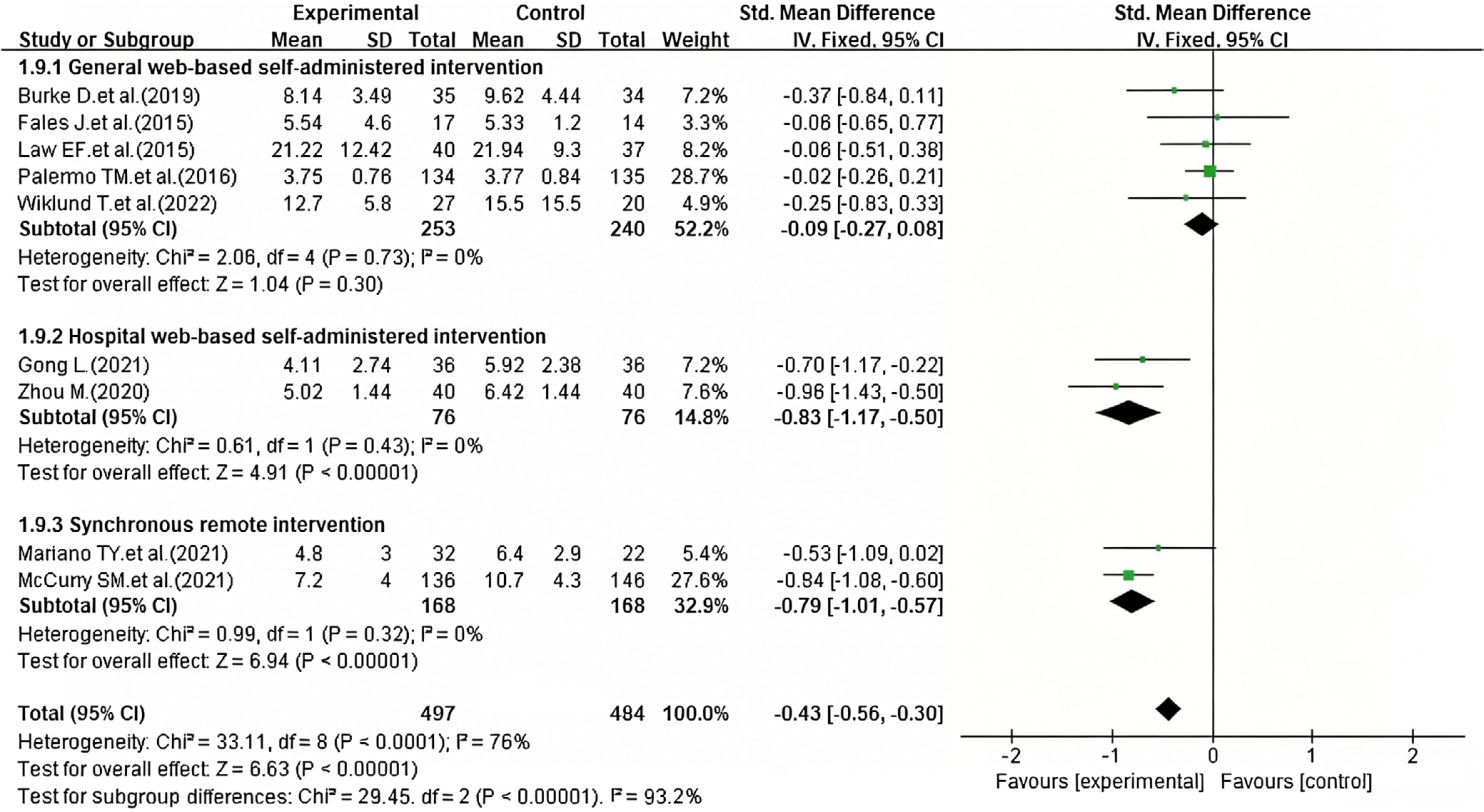
Forest plot of subgroup analysis: Efficacy of iCBT on sleep quality by intervention delivery modes. The horizontal axis represents the standardised mean difference (*SMD*) for sleep quality; negative values indicate better sleep quality in the iCBT group compared to the control group.

The pooled effect of interventions on sleep quality in patients with chronic pain showed high heterogeneity across all included studies (*I* ^2^ = 76%, *p* < 0.0001), thus, a random‐effects model was used for the overall meta‐analysis. To explore the sources of this substantial heterogeneity, subgroup analyses were performed by intervention delivery mode (general web‐based self‐administered, hospital web‐based self‐administered, and synchronous remote intervention). Of the nine included studies, five were general web‐based self‐administered interventions (Burke et al. 2019; Fales et al. 2015; Law et al. 2015; Palermo et al. 2016; Wiklund et al. 2022), two were hospital web‐based self‐administered interventions (Gong 2021; Zhou 2020), and two were synchronous remote interventions (Mariano et al. 2021; McCurry et al. 2021). Subgroup analysis by intervention delivery mode revealed that all three subgroups exhibited no heterogeneity (*I* ^2^ = 0% for each), allowing the use of a fixed‐effect model for each subgroup: for the general web‐based self‐administered interventions, the results showed no statistically significant difference (SMD = −0.09, 95% CI: −0.27 to 0.08, *p* = 0.30) (Figure 10), suggesting no significant improvement in sleep quality; for the hospital web‐based self‐administered interventions, the results demonstrated a statistically significant difference (SMD = −0.83, 95% CI: −1.17 to −0.50, *p* < 0.0001) (Figure 10), indicating a significant improvement in sleep quality, and for the synchronous remote interventions, the results also showed a statistically significant difference (SMD = −0.79, 95% CI: −1.01 to −0.57, *p* < 0.0001) (Figure 10), confirming a significant improvement in sleep quality.

## 4 Discussion

### 4.1 Pain Symptoms

This study incorporated nine relevant articles, with a total sample size of 998 patients. The meta‐analysis results demonstrate that iCBT positively impacts pain symptoms in patients with chronic pain. This finding is consistent with the findings of (Gandy et al. 2022). Five of the included studies had follow‐up assessments. The meta‐analysis of intergroup differences in pain scores after long‐term follow‐up and post‐intervention data revealed no statistically significant differences. This suggests that iCBT demonstrated significant short‐term pain improvement, but its long‐term effects are limited and did not reach statistical significance, in agreement with the findings of G. Andersson (Andersson et al. 2018; Van der Zweerde et al. 2020). Moreover, the meta‐analysis results indicate that although the intervention group showed improvements in pain during the follow‐up period, the degree of improvement did not achieve statistical significance. This reflects the long‐term challenges in patients with chronic pain treatment—even psychological interventions face difficulties in sustaining long‐term relief. Several factors may contribute to this phenomenon. First, the persistence and complexity of chronic pain suggest that a single psychological intervention may not be effective over the long term. Many chronic pain patients experience a rebound in pain after treatment, particularly in the absence of continuous support. Second, the treatment of chronic pain frequently involves a combination of approaches, including medication, physical therapy, and psychological interventions. A single iCBT intervention may not be sufficient to sustain long‐term effects, particularly when patients fail to continue engaging in cognitive‐behavioural practices in daily life. Nonetheless, iCBT continues to offer some sustained benefits for chronic pain patients. Four of the five follow‐up studies indicated that, after the follow‐up period, the pain levels in the intervention group remained lower than those in the control group, suggesting that although iCBT's effects diminished over time, the improvements may still possess clinical relevance. This finding implies that although iCBT may not sustain significant therapeutic effects over the long term, it still provides patients with tools to better manage their pain as a short‐term supplementary intervention rather than a long‐term curative measure. By adjusting cognitions, modifying coping strategies, and enhancing emotional regulation skills, patients can maintain a certain level of pain relief after treatment, particularly in the absence of strong pharmacological interventions. These potential sustained benefits are closely tied to the mechanisms of action of iCBT. iCBT helps patients identify and modify negative thinking patterns and cognitive distortions related to pain, improving catastrophic thinking and promoting more adaptive coping strategies, thereby reducing the perception of pain (Selvanathan et al. 2021). This treatment approach may not only influence patients' thought patterns but also alter their pain experience through physiological mechanisms. For instance, research indicates that iCBT can reduce brain activity related to pain, particularly in regions associated with emotional processing, such as the prefrontal cortex and amygdala (Brooks and Stein 2015; Sallet et al. 2013). These changes in neural mechanisms may contribute to pain relief, further supporting the effectiveness of iCBT in patients with chronic pain management. It should be emphasised that the long‐term effects of iCBT on pain did not reach statistical significance. This finding may be related to factors such as study intervention duration and patient adherence. Therefore, its sustained benefits need to be cautiously interpreted in clinical practice. It is recommended to combine iCBT with continuous follow‐up management, medication, and physical therapy to achieve the synergistic consolidation of short‐term pain improvement effects and the optimisation of long‐term efficacy.

### 4.2 Sleep Quality

In the meta‐analysis of iCBT for improving sleep quality in patients with chronic pain, statistically significant differences between the intervention and control groups were observed, suggesting the effectiveness of the intervention. However, the included studies demonstrated high heterogeneity. To explore the sources of this heterogeneity, subgroup analyses were conducted based on four factors: whether patients were diagnosed with insomnia, the type of sleep assessment tool used, age (age ≥ 18 years), and the intervention delivery modes. The results indicated that iCBT significantly improved sleep quality in patients with chronic pain, regardless of whether they were diagnosed with insomnia. This finding is consistent with both domestic and international research (Lin et al. 2024; Zachariae et al. 2018). This further validates iCBT as a flexible and effective intervention that can be widely applied across diverse populations, especially for those with mild insomnia symptoms or those not yet formally diagnosed. This finding is particularly relevant for chronic pain patients with sleep disorders, as they often experience sleep disturbances due to ongoing pain, including difficulty falling asleep, frequent awakenings, or poor sleep quality (Barazzetti et al. 2022; Finan et al. 2013). However, despite these significant sleep disturbances, the proportion of patients diagnosed with insomnia remains relatively low. This is because sleep disturbances caused by chronic pain often fall into a ‘suboptimal health’ state, where sleep quality deteriorates significantly but does not meet the diagnostic criteria for insomnia. Consequently, many patients' sleep disturbances are not adequately assessed, and timely intervention is often lacking, leading to a vicious cycle of poor sleep and pain. This suggests that healthcare providers should enhance the early recognition and assessment of sleep disturbances in pain management and increase the use of cognitive behavioural therapy interventions, such as sleep‐focused cognitive behavioural therapy, to break the vicious cycle between sleep disorders and pain, thereby improving overall health outcomes.

Furthermore, the subgroup analysis based on sleep assessment tools demonstrated statistical significance with the Insomnia Severity Index (ISI) and the Athens Insomnia Scale in evaluating the effectiveness of iCBT, which is consistent with the literature (Lin et al. 2020; Morin et al. 2011). These two tools exhibit high reliability and validity in assessing sleep quality (Enomoto et al. 2018; Gagnon et al. 2013) and can accurately reflect the severity of sleep disturbances and improvements. In contrast, the subgroup analysis using the Numeric Rating Scale (NRS) did not show statistical significance, possibly due to the NRS being a unidimensional scale, which may not be sensitive enough to detect subtle changes in sleep improvement.

Future researchers should prioritise the adoption of validated, high‐sensitivity multi‐dimensional scales (e.g., ISI, AIS, and BPI) and, in light of study design and objectives, combine multiple sleep or pain assessment tools to avoid the limitations of single‐dimensional evaluation, thereby comprehensively capturing sleep‐related and pain‐related outcomes from multiple dimensions. Meanwhile, integrating objective assessment methods (e.g., polysomnography (PSG), sleep duration recorded by wearable devices) with subjective scales can further enhance the robustness and credibility of outcome data. Building on this foundation, future studies on sleep improvement should also integrate subjective and objective assessment results to dynamically monitor intervention effects, thus revealing sleep improvement outcomes more comprehensively and accurately.

Then, the subgroup analysis based on age revealed that iCBT was significantly effective for chronic pain patients aged ≥ 18 years, while patients under 18 did not show significant benefits. This difference may be attributed to the unique physiological needs, psychological development, and coping strategies of the adolescent group. Specifically, adolescents' immature cognitive development impairs their response to cognitive restructuring‐based iCBT (Garber et al. 2016). Their low adherence to web‐based self‐interventions is associated with academic pressure and inadequate parental supervision (Achilles et al. 2020). Furthermore, adult‐oriented iCBT protocols lack age‐appropriate elements (e.g., gamification), leading to poor engagement (Christie et al. 2019). Adolescents are in a critical period of growth and development, and factors such as academic pressure, lifestyle, and physiological changes make them more susceptible to insomnia in the context of chronic pain (Sidani et al. 2021). Moreover, adolescents often have lower acceptance of CBT, as they tend to rely more on social and emotional support rather than independently engaging in cognitive adjustments (Rasing et al. 2021). Peersmann suggested that CBT‐I has a significant short‐term effect in adolescents, but its long‐term effects are less pronounced compared to adults (Peersmann et al. 2021). Future studies should develop adolescent‐specific iCBT protocols with gamified modules and parental involvement to improve intervention adherence. To address this issue, nursing interventions should be tailored to adolescents' psychological characteristics and behavioural needs, enhancing adherence through engaging and interactive educational content. Additionally, encouraging the involvement of parents and schools in creating a supportive environment is important. Finally, healthcare providers should establish a continuous follow‐up feedback mechanism, promptly adjusting intervention plans, and helping adolescents gradually develop regular sleep habits to enhance the effectiveness of iCBT in alleviating pain and improving sleep.

Finally, a subgroup analysis of intervention delivery modes was conducted in this study. The results demonstrated that hospital‐specific web‐based interventions and synchronous remote interventions (e.g., online conferences) exhibited statistically significant therapeutic efficacy, whereas general web‐based self‐administered interventions failed to show statistical significance. This disparity in efficacy may be attributed to the variations in supervision levels across different delivery modes: hospital‐specific platforms can enhance patients' trust in and adherence to the intervention, and real‐time remote guidance not only ensures the proper implementation of cognitive behavioural skills but also guarantees the standardisation of intervention delivery through external supervision. In contrast, unsupervised self‐administered interventions are highly dependent on patients' own self‐management abilities, making them prone to poor intervention adherence and inadequate implementation standardisation. This finding is consistent with the conclusions of previous studies that guided digital interventions yield superior efficacy in patients with chronic diseases (Musiat et al. 2022). In clinical practice, nursing staff may select iCBT delivery modes for patients with chronic pain in a targeted manner based on their self‐management abilities: for patients with poor adherence, guided intervention modes (e.g., hospital‐specific web‐based interventions and synchronous remote guidance) should be prioritised; for those receiving self‐administered interventions, enhanced regular follow‐up and online guidance are required to ensure the therapeutic efficacy of iCBT.

## 5 Strengths and Limitations

### 5.1 Review Strengths

#### 5.1.1 Comprehensiveness and Scope

This study systematically evaluates the effects of iCBT on chronic pain and sleep quality by synthesizing multiple randomised controlled trials (RCTs). The study incorporates a wide range of studies and sample populations, with a relatively large total sample size, thereby enhancing the generalizability and reliability of the findings.

#### 5.1.2 Strong Clinical Relevance

The results of this study demonstrate that iCBT not only effectively alleviates chronic pain but also significantly improves sleep quality, particularly among adults aged ≥ 18 years. These findings provide strong clinical implications, offering a non‐pharmacological treatment option for chronic pain sufferers, especially for those who lack access to conventional therapeutic resources.

#### 5.1.3 Broad Subgroup Analysis

The study conducted a subgroup analysis of sleep improvement, which included both diagnosed and undiagnosed insomnia patients, as well as adults from various age groups. This comprehensive analysis provides practical guidance for the clinical application of iCBT across different patient subgroups.

### 5.2 Review Limitations

#### 5.2.1 Heterogeneity of the Study Samples

Although this study included multiple randomised controlled trials (RCTs), there were variations in the iCBT interventions employed across the trials (e.g., treatment duration, intervention content). The interventions varied in terms of duration, therapist involvement, and degree of personalisation, with some studies offering fully automated treatments and others incorporating therapist support. In addition, different assessment tools were used to evaluate pain symptoms and sleep quality across the included studies, and while all interventions were classified as short‐term, their durations varied widely from 2 to 10 weeks, which may have compromised the consistency of intervention effects. Furthermore, the included patient populations exhibited marked diversity in key characteristics such as age, comorbidity status, and chronic pain severity, all of which are important contributors to the overall heterogeneity of the study. Future research should standardise intervention protocols, adopt unified assessment tools and fully account for differences in patients' core characteristics to minimise the impact of heterogeneity on study results.

#### 5.2.2 Short Follow‐Up Period and Unclear Long‐Term Effects

Some of the studies included in this research lacked long‐term follow‐up data, with only five studies including follow‐up data and varying follow‐up intervals. This limitation prevents a comprehensive evaluation of the long‐term effects of iCBT on chronic pain and sleep quality. Future research should extend the follow‐up period to assess the sustainability of treatment effects and recurrence rates.

#### 5.2.3 Geographical Limitations of the Study Sample

The samples in the current study mainly came from several countries in Europe, the United States, and Asia, which may not fully represent chronic pain patients from other regions, particularly those in low‐income countries. Future studies should include a broader range of geographic locations to examine the applicability and effectiveness of iCBT in diverse cultural and economic contexts.

## 6 Conclusion

This study systematically assessed the effects of iCBT on chronic pain and sleep quality improvement through a meta‐analysis of multiple randomised controlled trials (RCTs). The results indicated that iCBT significantly reduced chronic pain symptoms in the short term and effectively improved sleep quality, particularly among adults aged ≥ 18 years. As a non‐pharmacological intervention, iCBT demonstrated strong clinical applicability, especially for chronic pain patients who have limited access to traditional treatment resources, serving as a supplementary treatment. However, several limitations were identified in this study, including sample heterogeneity, a relatively short follow‐up period, and geographical constraints. Notably, iCBT's long‐term therapeutic effect on chronic pain is limited. Future research should focus on standardizing intervention protocols, extending follow‐up durations to evaluate the long‐term effects of iCBT, and broadening the study population to include diverse geographical regions and cultural backgrounds in order to further validate its global effectiveness and applicability.

In conclusion, iCBT provides an effective non‐pharmacological approach for the short‐term management of chronic pain and improvement of sleep quality. While its short‐term analgesic effects are promising, its long‐term efficacy is limited, as highlighted by the meta‐analysis findings. Additional studies are needed to explore iCBT's long‐term effects, refine its protocols, and confirm its applicability across diverse patient populations.

## Author Contributions

Yuxin Liu: Conceptualisation, Data curation, Formal analysis, Methodology, Validation, Writing – original draft, Writing – review and editing. Hong Ji: Methodology, Supervision, Validation, Writing – review and editing. Saiwei Bai: Data curation, Methodology, Validation, Writing – review and editing. Xueqing Tian: Formal analysis, Writing – review and editing. Wenzhong Zhang: Formal analysis, Writing – review and editing. Guoan Jia: Data curation, Writing – review and editing.

## Funding

The authors have nothing to report.

## Conflicts of Interest

The authors declare no conflicts of interest.

## Data Availability

All data used in this meta‐analysis were extracted from previously published studies and are available in the respective original articles. No new datasets were generated in the present study.

## Appendix 1

**Table:**

Database	Search Strategy
PubMed	((“Web‐assisted” [Mesh]OR “Web‐based” [Title/Abstract] OR “Internet” [Title/Abstract] OR “Online” [Title/Abstract] OR “Computer‐based” [Title/Abstract] OR “Website” [Title/Abstract] OR “Smartphone” [Title/Abstract] OR “Mobile Application” [Title/Abstract] OR “mHealth” [Title/Abstract] OR “eHealth” [Title/Abstract] OR “Telehealth” [Title/Abstract])) AND (“Cognitive Behavioural Therapy” [Mesh] OR “Cognitive Behavioural Therapy” [Title/Abstract] OR “CBT‐I” [Title/Abstract] OR “Internet‐based Cognitive Behavioural Therapy” [Title/Abstract] OR “Online Cognitive Behavioural Therapy” [Title/Abstract]) AND ((“Chronic Pain” [Mesh] OR “Chronic Pain” [Title/Abstract] OR “Pain, Chronic” [Title/Abstract]))
Web of Science	TS = (Web‐assisted or telephone or Web‐based or Internet Online or Computer‐based or Website or Smartphone or Mobile Application or mHealth or eHealth or Telehealth)AND TS = (Cognitive Behavioural Therapy or CBT‐I or Internet‐based Cognitive Behavioural Therapy or Online Cognitive Behavioural Therapy) AND TS = (chronic pain or chronic pain*)
Embase	(('web‐assisted'/exp. OR 'web‐assisted':ti,ab,kw OR 'web‐based'/exp. OR 'web‐based':ti,ab,kw OR 'internet'/exp. OR 'internet':ti,ab,kw OR 'online'/exp. OR 'online':ti,ab,kw OR 'computer‐based'/exp. OR 'computer‐based':ti,ab,kw OR 'website'/exp. OR 'website':ti,ab,kw OR 'smartphone'/exp. OR 'smartphone':ti,ab,kw OR 'mobile application'/exp. OR 'mobile application':ti,ab,kw OR 'mhealth'/exp. OR 'mhealth':ti,ab,kw OR 'ehealth'/exp. OR 'ehealth':ti,ab,kw OR 'telehealth'/exp. OR 'telehealth':ti,ab,kw) AND ('cognitive behavioural therapy'/exp. OR 'cognitive behavioural therapy':ti,ab,kw OR 'cbt‐i':ti,ab,kw OR 'internet‐based cognitive behavioural therapy':ti,ab,kw OR 'online cognitive behavioural therapy':ti,ab,kw) AND ('chronic pain'/exp. OR 'chronic pain':ti,ab,kw OR 'pain, chronic'/ti,ab,kw))
Cochrane Library	“Web‐assisted” OR “Web‐based” OR “Internet” OR “Online” OR “Computer‐based” OR “Website” OR “Smartphone” OR “Mobile Application” OR “mHealth” OR “eHealth” OR “Telehealth” (All Fields) AND “Cognitive Behavioural Therapy” OR “CBT‐I” OR “Internet‐based Cognitive Behavioural Therapy” OR “Online Cognitive Behavioural Therapy” (All Fields) AND “Chronic Pain” OR “Pain, Chronic” (All Fields)
CNKI (中国知网)	SU = "网络辅助" + "基于网络" + "互联网"+"在线"+"计算机辅助" +"网站" +"智能手机" +"移动应用"+"移动健康"+"电子健康" +"远程医疗"+"计算机辅助" AND SU = "认知行为疗法" +"失眠认知行为疗法" +"基于互联网认知行为疗法" +"在线认知行为疗法" AND SU = "慢性疼痛"+"疼痛"+"持续性疼痛慢性"+"肌肉骨骼疼痛"
Wanfang Database	(网络辅助 OR 基于网络 OR 互联网 OR 在线 OR 计算机辅助 OR 网站 OR 智能手机 OR 移动应用 OR 移动健康 OR 电子健康 OR 远程医疗) AND (认知行为疗法 OR 失眠认知行为疗法 OR 基于互联网认知行为疗法 OR 在线认知行为疗法) AND (慢性疼痛 OR 疼痛 OR 持续性疼痛 OR 慢性肌肉骨骼疼痛)
VIP Database	(网络辅助 OR 基于网络 OR 互联网 OR 在线 OR 计算机辅助 OR 网站 OR 智能手机 OR 移动应用 OR 移动健康 OR 电子健康 OR 远程医疗) AND (认知行为疗法 OR 失眠认知行为疗法 OR 基于互联网认知行为疗法 OR 在线认知行为疗法) AND (慢性疼痛 OR 疼痛 OR 持续性疼痛 OR 慢性肌肉骨骼疼痛)
